# Indoor Air Quality Issues for Rocky Mountain West Tribes

**DOI:** 10.3389/fpubh.2021.606430

**Published:** 2021-03-05

**Authors:** Logan Webb, Darrah K. Sleeth, Rod Handy, Jared Stenberg, Camie Schaefer, Scott C. Collingwood

**Affiliations:** ^1^Department of Family and Preventive Medicine, Rocky Mountain Center for Occupational and Environmental Health, University of Utah, Salt Lake City, UT, United States; ^2^Physician Assistant Studies, Department of Family and Preventive Medicine, University of Utah, Salt Lake City, UT, United States; ^3^Department of Pediatrics, University of Utah, Salt Lake City, UT, United States

**Keywords:** Native American, community-based participatory research, environmental health, tribal housing, radon, PM2.5

## Abstract

Native American populations face considerable health disparities, especially among those who live on reservations, where access to healthcare, education, and safe housing can be limited. Previous research on tribal housing has raised concerns about housing construction, damage, and possible linkage to adverse health effects (e.g., asthma). This community-based participatory research (CBPR) project investigated indoor air quality issues on two Rocky Mountain west reservations. At the onset of the project, the research team formed a partnership with community advisory boards (CABs) consisting of representatives from tribal councils and community members. Research design, implementation, and dissemination all took place in full collaboration with the CABs following approval through official tribal resolutions. Residential homes were monitored for particulate matter with diameter <2.5 microns (PM_2.5_) and radon concentrations. Low-cost air quality sensors and activated charcoal radon test kits were placed in tribal households for 6-8 days. A large amount of data were below the sensor limit of quantification (LOQ), but several homes had daily averages that exceeded suggested PM2.5 guidelines, suggestive of the potential for high exposure. Additionally, nearly half of all homes sampled had radon levels above the EPA action level, with mitigation activities initiated for the most concerning homes. Findings from this study indicate the need for future community-wide assessments to determine the magnitude and patterns of indoor air quality issues.

## Introduction

Native American populations face considerable health disparities, including a lower life expectancy and higher rates of chronic illnesses such as diabetes, cardiovascular diseases, and certain cancers (1). Researchers have identified socioeconomic status and environmental injustice factors of inadequate housing, limited access to healthcare, and lack of education as contributing factors to such health disparities (1). Of particular interest here is the fact that many Native Americans do not have access to affordable and safe housing, particularly on reservations, and live in insufficiently built and/or deteriorated housing (2, 3). A 1950's Housing and Urban Development (HUD) initiative that sought to construct standardized housing on reservations resulted in much of today's tribal housing being poorly suited for local climates and therefore susceptible to mold and other indoor air quality issues (4). Housing-related disparities such as inadequate plumbing and ventilation have been linked to environmental health, including exposures to lead, allergens, pesticide residue, and other issues (5). Furthermore, much of tribal housing relies on wood-burning and/or unrefined coal as a primary heating/cooking source, which in turn exacerbates issues of indoor air quality.

Indoor air quality is of paramount concern for human health, since people can spend up to 90% of their time indoors (6). There is currently no indoor particulate matter (PM) exposure standard, although the World Health Organization (WHO) suggests that existing outdoor air quality standards are potentially applicable (7), e.g., the Environmental Protection Agency (EPA) 24-h outdoor PM2.5 standard of 35 μg/m^3^ (8). Poor air quality has been associated with heightened mortality and morbidity in numerous studies (9–11). In particular, PM2.5 (PM < 2.5 μm in size) air pollution has been a focus of study as a contributor to disease. PM2.5 is primarily generated by combustion sources (i.e., wood-burning, coal, gasoline, etc.) and is small enough to penetrate deeply into the lungs, leading to potential long-term damage to the respiratory system (11, 12).

Chronic exposure to PM2.5 has been linked to increased rates of cardiovascular disease, pulmonary disease, and respiratory outcomes such as aggravated asthma and decreased lung function, all of which may culminate in early mortality (10, 11, 13–15). Increasing evidence has found other potential associations with PM2.5 exposure and other health issues, including the development of neurodegenerative disorders such as dementia (16, 17) and loss of reproductive function (18, 19). Vulnerable populations, including children, the chronically ill, and the elderly are particularly at risk due to increased particulate pollution (20–24).

Cardiopulmonary and respiratory illnesses are seen at dramatically higher rates among Native American tribes. Native populations experience a 20% higher mortality rate due to cardiovascular disease compared to the general population (25). Native American children are 60% more likely to be diagnosed with asthma than white non-Hispanic children (26), and are hospitalized for lower respiratory tract infections at a rate 1.5 times greater than the general population (27).

Radon is another major source of indoor air pollution linked to poor-quality housing and conducive geological conditions (e.g., cracks in the foundation, contaminated well water, zonal elevated radon concentrations) (28). Radon, a dense gas that forms from the natural radioactive decay of uranium, thorium, and radium in the earth's crust, rises to hazardous levels only in enclosed buildings (29, 30). This is of particular concern in the western U.S., where naturally occurring uranium in the ground creates an increased potential for high indoor levels of radon (31). However, radon exposure in tribal populations are not well-known outside of a few limited studies, such as the Navajo Nation Radon Program (32). The EPA estimates that radon is responsible for more than 20,000 lung cancer deaths in the U.S. each year, and is the second most common causal factor outside of tobacco smoking (30, 33). Lung cancer is the leading cause of cancer death among Native American populations, and while this is generally attributed to higher rates of tobacco use, growing concerns have been expressed regarding the role of radon exposure (34, 35). Of particular concern are observations regarding the synergistic effect of radon and tobacco smoke on lung cancer rates (36).

A number of studies have examined elements of indoor air pollution in Native American homes, including wood-burning stoves (37), tobacco smoke (38), and outdoor air contamination from nearby anthropogenic sources (e.g., oil/gas extraction, mining, nearby industry) (39, 40). However, the literature is limited to the specific exposures, and particular challenges, of a small number of tribes. In particular, frontier (i.e., extremely rural) Native American reservations with smaller populations have been overlooked in favor of larger tribes and more readily accessible reservations (41). This gap in research can also be partially attributed to a history of exploitative research on Native American populations, which has created barriers to the conduct of research (42). In order to begin addressing this dearth of research, this project partnered with **two** frontier reservations in the Rocky Mountain West to conduct community-based participatory research (CBPR) that focused on key indoor air quality screening data. The goal was to **first** assess local indoor air quality, and then to inform the tribal communities of any concerning levels in need of mitigation and to establish recommendations for future assessments.

## Methods

Two distinct tribes (referred to as Tribe A and B) located in the Mountain West region of the United States were surveyed for this study. In both cases, tribal community leaders and researchers formed a CBPR plan that sought input from stakeholders throughout the process, including study design, recruitment, implementation, and dissemination. The first step in the CBPR process was the establishment of a community advisory board (CAB) for each tribe. The CAB was composed of various tribal leaders (e.g., tribal council members and heads of local housing, environmental, and health departments) and community members (e.g., elders, parents, and teachers). Conversations with each CAB helped identify environmental areas of concern for each tribe. Indoor air quality was selected as a priority concern, as there was a perception of a high prevalence of respiratory conditions (e.g., asthma) within each tribe. Additional concerns regarding air quality stemmed from a history of environmental spoliation within each tribe, including local mining activities, known uranium deposits, and other anthropogenic sources.

Following study conceptualization with the CAB, support and participation from tribal programs (e.g., housing, environment, and health) was obtained and public presentations on the study objectives were conducted as part of the CBPR process. The research team then brought the study to the respective tribal councils for approval, and with the help of CAB advocates, tribal resolutions were approved by each tribe, which enabled the research to commence. It was agreed with each tribe that they would remain owners of the data and that both the tribal identities and those of individual participants would be de-identified in publications and presentations. However, throughout the research, tribal partners were present and engaged with participant recruitment, home visits, data interpretation, disseminating results to the wider community, and planning future work. Each tribe had specific tribal members who were the primary points of contact on each reservation, helping to coordinate activities and sustain the CBPR approach.

Recruitment took place in February/March 2018 (Tribe A) and in November 2018 (Tribe B). Prior to active recruitment, the University of Utah IRB determined this was “exempt” research, which was determined to be sufficient by the tribes and therefore no tribal IRBs were consulted. Tribe members were given study information to learn more about the research and what participation would entail. Scheduled meetings for recruitment were subsequently held at local community centers, where researchers explained health risks associated with poor indoor air quality. Researchers demonstrated the air sampling equipment and a question-and-answer period allowed prospective participants to discuss any concerns. Participating households were required to (1) provide consent to monitor air quality at their home (from an adult resident who could voluntarily provide access to the home), and (2) have at least one adult (>18 years old) residing in the home full time. Participants were compensated by gift cards to cover their time (2 home visits) and use of electricity.

Houses were recruited from different geographic communities on each reservation. A total of 11 households were recruited from Tribe A (representing ~10% of homes on the reservation) and 8 households from Tribe B (~0.5% of tribal member homes). All sampling took place in March/April 2018 (Tribe A) and November 2018 (Tribe B). A previously validated dwelling unit survey (43) was conducted at each house with the help of an adult household member. These surveys were not incorporated into data analysis, but were reviewed for relevant data regarding housing safety and health and then archived for future community or study use.

The instrument chosen to monitor indoor PM concentrations in this study was the AirU, a low-cost integrated sensor developed by the University of Utah that utilizes the Plantower PMS 3330 laser particle counter to measure PM2.5 exposures (44, 45). The device took measurements every 15 s, which were then averaged to give 1-min exposure estimates. This averaging is a design feature of the AirU intended to limit the number of data points for sampling that occurs over days, weeks, or months. Data were logged on a microSD card. Each AirU sensor was calibrated prior to deployment and assigned a calibration equation specific to that sensor (46). For calibration, sensors were placed in a cylindrical chamber with aerosolized ammonium nitrate, alongside a reference-grade instrument (Dustrak DRX) (45). The raw data collected as part of this study was corrected using the assigned calibration equation established for each individual sensor. The limit of quantification (LOQ) for the sensor was previously determined to be ~5 μg/m^3^ (45).

The number of sensors installed at each home was based on device availability on the day of installation. In each home, at least one AirU sensor was deployed in what was identified by a member(s) of the household as the most frequently used room by a member of the household. If equipment was available, two sensors were installed inside a home to obtain a more comprehensive understanding of indoor air quality, with the second device placed in the main bedroom. A subsection of homes also had outdoor sensors deployed in addition to indoor sensors to better understand the relationship between indoor and outdoor air quality for a given home.

Sensors were installed by members of the research team, who ensured that the instrument was placed on a flat surface and correctly oriented. The sensors were placed off the ground to ensure they were at a relevant height (e.g., at mouth level) and in places least likely to be disturbed. Sensors were in place for 6 days in Tribe A homes and 8 days in Tribe B homes.

All homes that participated in PM2.5 sampling were also monitored for radon, which was measured by activated-charcoal passive test kits, which were in place for 5–7 days. The EPA recommends placing test kits on the lowest-level sleeping area of the home (47), so test kits were placed accordingly, with the exception of one home that had a main bedroom on the second floor. The test kits were either hung ~0.5 m from the ceiling or placed on a flat surface 1–1.5 m off the ground. This placement ensured that sampling primarily took place at the height of a typical human breathing zone (i.e., nose/mouth). All test kits were placed in the center of the room as best as possible. After completion of radon sampling, the test kits were sealed with a self-adhesive strip on the package and immediately sent overnight to the accredited Air Chek Laboratory (Mills River, North Carolina) to be analyzed. Analysis typically occurred the same day the test was received by the laboratory.

Descriptive statistics and graphical analyses were performed for each AirU sensor. The proportion of values below the LOQ were also calculated. Homes were excluded from analysis if there were missing or corrupted PM2.5 data due to sensor malfunction. All data were analyzed using Excel (Microsoft, Redmond, Washington).

## Results

Table 1 provides the PM2.5 and radon concentration results for both tribes. Every home in Tribe A used a wood-burning stove for heating, with two homes having visual signs of cigarette smoking. All homes surveyed in Tribe B used electric or natural gas devices for heating and cooking, with no observed cigarette smoking.

**Table 1:** PM2.5 and radon concentrations for Tribe A and B residential homes.

	**PM2.5**			**Radon**		
	**No. of samples**	**Peak concentration (μg/m**^**3**^**)**	**Percent** **<** **LOQ**	**No. of homes**	**Geometric Mean (pCi/L)**	**Geometric standard deviation**
Tribe A	77,549 (*n* = 8 homes)	463	87.6	11	3.9	2.6
Tribe B	142,164 (*n* = 7 homes)	896	82.8	8	3.8	1.9

Sensor malfunctions led to the exclusion of all PM2.5 data in 3 of the 11 homes for Tribe A and 1 out of 8 for Tribe B. Over 80% of the PM2.5 data collected from both tribes were below the LOQ (Tribe A: 87.6%; Tribe B: 82.8%). For Tribe A, the amount of data < LOQ ranged from 45 to 99% for individual homes; for Tribe B, it ranged from 29 to 97% for individual homes. The peak PM2.5 concentration was nearly twice as high for Tribe B (896 μg/m^3^) than Tribe A (463 μg/m^3^). Two homes in Tribe B had at least one average daily exposure concentrations above 35μg/m^3^, one house with 6/8 days and one with 5/8 days above that level (see Table 2).

**Table 2:** Tribe B daily concentration averages in μg/m^3^ for each indoor sensor (LOQ = 4.8 μg/m^3^).

**House/Sensor No**.	**Day 1**	**Day 2**	**Day 3**	**Day 4**	**Day 5**	**Day 6**	**Day 7**	**Day 8**
**1**								
*Sensor 1*	<LOQ	9.2	<LOQ	<LOQ	12.8	<LOQ	9.5	<LOQ
*Sensor 2*	<LOQ	31.8	<LOQ	<LOQ	23.6	<LOQ	15.5	<LOQ
**2**								
*Sensor 1*	5.45	<LOQ	<LOQ	<LOQ	<LOQ	<LOQ	<LOQ	<LOQ
*Sensor 2*	7.37	<LOQ	<LOQ	<LOQ	7.21	<LOQ	<LOQ	<LOQ
**3**								
*Sensor 1*	6.7	**51.9**	28.1	15.1	18.2	34.4	**54.2**	6.8
*Sensor 2*	26.4	**93.7**	**56.9**	**72.1**	**84.1**	**61.2**	**120.9**	10.0
**4**								
*Sensor 1*	26.0	**69.6**	22.9	**59.5**	**47.0**	**44.3**	**46.6**	18.9
*Sensor 2*	15.5	**59.0**	11.8	**103.3**	**52.0**	**57.1**	**85.4**	24.0
**5**	<LOQ	<LOQ	<LOQ	<LOQ	<LOQ	<LOQ	<LOQ	<LOQ
**6**	<LOQ	12.9	18.3	5.1	15.9	7.5	<LOQ	<LOQ
**7**								
*Sensor 1*	<LOQ	<LOQ	<LOQ	<LOQ	<LOQ	<LOQ	<LOQ	<LOQ
*Sensor 2*	<LOQ	<LOQ	<LOQ	<LOQ	<LOQ	<LOQ	<LOQ	<LOQ

*Values in bold exceeded the EPA 24-h PM2.5 standard (35 μg/m^3^)*.

No radon samples were excluded from analysis. For Tribe A, 5 out of 11 homes (45%) had radon concentrations above the EPA action level (4pCi/L), with a geometric mean of 3.9. For Tribe B, 4 out of 8 homes (50%) were above that level, with a geometric mean of 3.8. The highest radon concentrations occurred in Tribe A homes (22.3 and 10.2pCi/L).

## Discussion

This study investigated indoor air pollution for two distinct tribal populations as part of a CBPR approach. The goal was to obtain a community-wide assessment that would inform further research and exposure mitigation (if warranted). While this study was somewhat limited by instrumentation malfunctions and sample size, the findings overall indicate a general trend toward air quality issues, particularly regarding radon, among the tribal homes measured, suggesting there is a need for remediation, and further sampling.

High levels of PM2.5 were generally not persistent within individual homes, which is suggested by the finding that <20% of the PM2.5 measurements were above the LOQ, despite relatively high peak concentration values. Briefly elevated levels may be due to individual living practices (e.g., cooking) and/or inadequacies in ventilation. However, intermittent spikes in PM2.5 have been linked to adverse reactions in sensitive populations (e.g., people with respiratory illnesses). Tribe B had somewhat more samples above the LOQ than Tribe A, suggesting overall higher levels of PM2.5 among homes in Tribe B.

Two homes within Tribe B had average readings that exceeded the EPA 24-h outdoor PM2.5 standard on multiple days, suggesting that problems with indoor air quality are worthy of further investigation. On the other hand, several houses had no exposures above the LOQ, which suggests that exposure, and therefore risk, may not be distributed evenly across tribal housing.

These findings were somewhat unexpected, as all homes in Tribe B were found to rely on non-combustion stoves (gas or electric), compared to Tribe A homes, which all used wood-burning stoves. High levels of PM2.5 within Tribe B homes are therefore likely due to other factors (e.g., cigarettes, candles, etc.). Future studies should analyze this information directly.

The time of year in which sampling was conducted (March/April for Tribe A, November for Tribe B) may also affect findings, as natural ventilation (i.e., open windows and doors) may be more frequently utilized during the warmer months. However, it should be noted that peak concentrations within both tribes were notably high, which suggests a variety of possible sources. For example, other studies have concluded wood-burning households experience higher concentrations of PM2.5 (48–50).

The mean radon concentration in both frontier communities was relatively high given the average radon concentration in the general U.S. population is 1.3pCi/L (51). Recommendations were made to the CABs and tribal councils that additional radon testing should be immediately prioritized. For Tribe A, two homes with the highest radon concentrations (22.3 and 10 pCi/L) were able to receive immediate remediation based on coordination of tribal leaders, researchers, and outside resources. This is a great example of the power of using the CBPR approach for environmental health research.

Since radon is a known carcinogen, there is believed to be no safe level of exposure. The EPA suggests that when household radon levels exceed 2pCi/L, follow-up testing should take place and remediation considered (51). The EPA also identifies three zones related to potential radon concentrations: Zone 1 is for counties with predicted screening levels >4 pCi/L, Zone 2 predicts levels to be between 2 and 4pCi/L, and Zone 3 <2pCi/L. Tribe A is located across several counties that are all either Zone 1 or 2, and Tribe B is located entirely in Zone 1. The consistent presence of radon in both tribes (nearly half in each home above the EPA action level) suggests a housing inequality concern, as within the general U.S. population only 1 in 15 homes are above the EPA action level (52). These results are of even greater significance given that Native Americans have a higher usage of tobacco products and there is a multiplicative effect between radon and smoking (36). Tribal authorities and leaders in environmental and housing departments were recommended to begin conducting radon confirmation sampling, testing in more homes, and remediation where needed.

The CBPR process, which has many advantages in ethnically diverse or minority communities (53), contributed to a real, tangible benefit for both tribes and laid a foundation for further research. The CBPR process uses existing resources within a community and encourages participation from individuals with a wide range of backgrounds to participate. This approach gives people in the community ownership of the research and the ability to determine what actions should be taken to accomplish their health goals (53). A direct benefit from the CBPR process is the ongoing effort to continue monitoring, collaboration, and education. Researchers remain engaged with both tribes to date. For Tribe A, a smaller tribe in the Mountain West, researchers have fostered a public-private partnership where continued radon testing (including confirmation testing) has resulted in ~50% of all tribal homes having undergone testing. Remediation has occurred in three homes and planned in several others that had confirmation tests >4 pCi/L. Relevant radon education and training are planned for tribal housing staff so they can continue testing and remediation in a sustainable manner, without having to rely on non-tribal entities.

For Tribe B, a larger tribe in the West, the initial pilot project engaged <1% of tribal homes. However, the pilot project and CBPR methodology resulted in continued engagement and the submission of a grant to a federal funding agency to scale up this project. The grant is premised on a continued partnership between tribal stakeholders and the research team, and plans for relevant interventions that leverage total ecological knowledge (TEK) and are conducted in a sustainable manner that will have a lasting impact on the tribe. Findings from this pilot study have illuminated new means of increasing community engagement and ownership of their environmental data. The current pandemic and the resultant reduction in personal interaction and travel between communities has hindered, but not halted, engagement and progress. It has, however, further reinforced the desire and necessity to foster tribal-centric activities and solutions to these environmental exposures and health concerns.

This study benefitted from the similarity of study methods between both tribes. However, the small sample size, relatively short duration of sampling (6–8 days), and a large proportion of data below the LOQ did not provide enough information to make conclusions on long-term PM2.5 exposures within either tribe. A sampling period of 2 months or longer would allow researchers to draw more conclusions on PM2.5 exposures. Ideally both short-term and long-term monitoring would be performed to cover multiple seasons and a variety of activities and occupant behaviors. Short-term monitoring is best for the assessment of acute exposures and long-term monitoring would help in assessing chronic exposures (54).

While there were limitations in the AirU device (most notably the high LOQ), its low cost and real-time data collection easily allowed the identification of peak PM2.5 concentrations across multiple locations simultaneously. Studies on the AirU sensor have shown a strong correlation (*r* > 0.85) with reference monitors in the lab and in ambient settings (44–46, 55, 56). One long-term field study characterized some strengths of the sensor (56), including good intra-sensor agreement over a year of deployment, but also identified seasonal differences in sensor response as a possible concern. Sayahi et al. (56) hypothesized that seasonal differences are the result of the physical characteristics of the predominant aerosol associated with seasons (e.g., secondary inorganic compounds in winter inversions compared to elemental carbon that predominates from a fall wildfire). As the focus of this work was on indoor air, such source differences may not be as relevant. However, future research would benefit from a more in-depth investigation into the seasonal differences in sensor response.

Future work on the AirU includes the development of real-time reporting of exposure levels to the user, which would allow occupants to see when spikes occur. This could potentially influence individual behavior by helping identify what events might have lead to the higher PM2.5 concentrations. Real-time reporting of results would be particularly beneficial to persons with asthma or other respiratory sensitivities, as it would allow them to observe concentration spikes in real time and change their environment or behavior. If AirU sensors were deployed in greater numbers for longer collection periods, the AirU could perhaps provide an even more comprehensive assessment of indoor and outdoor air quality within tribes. Targeted deployment of a reference instrument would further help validate these findings.

Future studies could also include more detailed interviews with residents, which would help determine confounding variables that might contribute to PM2.5 exposures and lay the groundwork for possible future interventions. In conjunction with additional environmental sampling, medical records of tribe members could also be obtained to identify relationships between PM2.5 exposures and cardio-respiratory illnesses. We are in talks with the larger of the two tribes to continue this work into that area. It should be noted that such studies are best conducted with medium to large size tribes to increase the likelihood of generating statistically valid sample sizes. These suggested future studies will require further investment into the CBPR approach, which will further empower and equip both tribes to control their exposures, determine how to best implement remediation, and enact healthy living practices among their tribe members.

## Conclusion

The CBPR approach to environmental health sampling on Native American reservations was shown to be successful for two distinct tribes. Overall, the PM2.5 data showed relatively low levels on average (>80% below the LOQ for both tribes), but with some concerning peaks over a week of exposure that could affect sensitive populations. The mean radon concentrations in both communities were above the US general population, with more than half of all homes above the EPA action level for radon. The CPBR approach helped quickly enable radon mitigation to begin where needed. Future studies should include a longer duration of PM2.5 measurements and the addition of real-time exposure feedback.

## Data Availability Statement

The raw data supporting the conclusions of this article will be made available by the authors, without undue reservation.

## Ethics Statement

Studies involving animal subjects: No animal studies are presented in this manuscript. Studies involving human subjects: This research was determined to be exempt by the University of Utah IRB. Inclusion of identifiable human data: No potentially identifiable human images or data is presented in this study.

## Author Contributions

RH, DS, and SC contributed to study design. LW, RH, DS, JS, and SC contributed to data collection. LW, RH, DS, JS, CS, and SC contributed to data analysis and manuscript preparation. All authors contributed to the article and approved the submitted version.

## Conflict of Interest

The authors declare that the research was conducted in the absence of any commercial or financial relationships that could be construed as a potential conflict of interest.
